# A comparison of freezer‐stored DNA and herbarium tissue samples for chloroplast assembly and genome skimming

**DOI:** 10.1002/aps3.11527

**Published:** 2023-06-05

**Authors:** Edward V. McAssey, Cassidy Downs, Mitsuko Yorkston, Clifford Morden, Karolina Heyduk

**Affiliations:** ^1^ School of Life Sciences University of Hawaiʻi at Mānoa Honolulu Hawaiʻi USA; ^2^ Department of Ecology and Evolutionary Biology University of Connecticut Storrs Connecticut USA

**Keywords:** cold storage, DNA bank, endemism, fragmentation, genomics, Hawaiian plants, herbarium

## Abstract

**Premise:**

The use of DNA from herbarium specimens is an increasingly important source for evolutionary studies in plant biology, particularly in cases where species are rare or difficult to obtain. Here we compare the utility of DNA from herbarium tissues to their freezer‐stored DNA counterparts via the Hawaiian Plant DNA Library.

**Methods:**

Plants collected for the Hawaiian Plant DNA Library were simultaneously accessioned as herbarium specimens at the time of collection, from 1994–2019. Paired samples were sequenced using short‐read sequencing and assessed for chloroplast assembly and nuclear gene recovery.

**Results:**

Herbarium specimen–derived DNA was statistically more fragmented than freezer‐stored DNA derived from fresh tissue, leading to poorer chloroplast assembly and overall lower coverage. The number of nuclear targets recovered varied mostly by total sequencing reads per library and age of specimen, but not by storage method (herbarium or long‐term freezer). Although there was evidence of DNA damage in the samples, there was no evidence that it was related to the length of time in storage, whether frozen or as herbarium specimens.

**Discussion:**

DNA extracted from herbarium tissues will continue to be invaluable, despite being highly fragmented and degraded. Rare floras would benefit from both traditional herbarium storage methods and extracted DNA freezer banks.

Biological collections are an essential part of biodiversity research (Lavoie, 2013). By preserving and making publicly available a variety of biological specimens, the broader community can accelerate progress on research and educational efforts. Herbaria are the main site of botanical collections and are often broadly accessible via digitization and databasing efforts (Soltis, 2017). Many botanical questions have been addressed by studying herbarium collections, including phenology (Lavoie and Lachance, 2006; Willis et al., 2017; Jones and Daehler, 2018), species distributions (Loiselle et al., 2007; Feeley, 2012), and trait diversity (Heberling, 2022). In addition to studies that leverage the morphological and location information provided by a herbarium specimen, there has been a growing appreciation of how herbarium tissue can be used in genomics studies to further our understanding of the evolutionary relationships and patterns found in these collections.

Because herbarium specimens by definition include their associated collection dates and locations, they can be used to document changes in genetic diversity, especially in species that are rare and/or endangered (Cozzolino et al., 2007; Rosche et al., 2022). In *Anacamptis palustris* (Jacq.) R. M. Bateman, Pridgeon & M. W. Chase, herbarium specimens were used to compare historical and present‐day patterns of genetic diversity (Cozzolino et al., 2007). Large phylogenomic studies now routinely incorporate DNA obtained from herbarium specimens (e.g., Zeng et al., 2018; Nevill et al., 2020; Jiang et al., 2022). In addition, herbarium specimens themselves can be sources of genetic diversity. For example, rare and endangered plant species can be grown from seeds derived from the fruit on herbarium sheets (Wolkis et al., 2022). While seed viability may be variable and species‐specific, it does still represent a potential conservation strategy (Godefroid et al., 2011).

Herbarium specimens are especially crucial in the study of rare, endemic floras that are often understudied and lack genomic resources. For example, of the 1400 plant species naturalized in the Hawaiian archipelago, 90% are endemic and nearly 400 are threatened or endangered (Department of Land and Natural Resources, 2023; Pacific Islands Fish and Wildlife Office, 2023). The development of genomic resources in diverse Hawaiian taxa would improve our understanding of adaptation, biogeography, and conservation in this unique flora. For example, the endemic ʻōhiʻa lehua (*Metrosideros polymorpha* Gaudich.) is a culturally and ecologically important species, holding a central role in both Hawaiian moʻolelo (legend) and in forests across the islands. By sequencing whole genomes of 131 individuals across 11 species in the genus, including ʻōhiʻa lehua, Choi et al. (2021) were able to draw inferences on the demographic history of the genus and the genomic extent of divergent selection in this adaptive radiation. In general, however, studies on the genomics of Hawaiian endemics are limited (but see Bellinger et al., 2022). Despite seemingly endless scientific interest in the radiation of species found in Hawaiʻi, endemic species in Hawaiʻi are often difficult to study due to their small population sizes and remote habitats. Herbaria are well suited for genomic investigations of species for which wild tissue collections are impractical from the perspective of cost, time, and/or safety. The ability to obtain sufficient DNA from a herbarium specimen can quickly jumpstart research on rare species.

Obtaining high‐quality DNA from herbarium specimens for downstream applications can be challenging (Savolainen et al., 1995; Staats et al., 2011; Särkinen et al., 2012), but has shown promise via sampling genomes using reduced representation approaches such as double‐digest restriction site–associated DNA sequencing (ddRADseq) in very old herbarium specimens with methods tested across four genera in Jordon‐Thaden et al. (2020). Hybrid capture protocols have also successfully produced large genomic data sets from relatively old herbarium samples (Hart et al., 2016). Additionally, Kates et al. (2021) showed that specimen age and extent of DNA degradation did not correlate with sequencing success, demonstrating that herbarium‐stored specimens of varying age can be used in phylogenomic studies. Despite considerable recent innovations in the area of herbarium DNA extractions, the effects of storage time, species, and their interaction can all limit the type of molecular work that is possible for a particular collection. To counteract the negative effects of time on a herbarium sheet, DNA can be extracted from the fresh tissue of the plant that is being accessioned (or a relative) and vouchered in cold storage while the plant tissue remains in the herbarium. This is the approach of the Hawaiian Plant DNA Library (HPDL), established in 1992 (Morden et al., 1996). Approximately 13,000 specimens have had their DNA extracted and stored at −20°C, representing approximately 89.7% of flowering plant genera and 92.7% of families that are found in Hawaiʻi. The collection also represents 49.3% of the genera and 70.4% of the families comprising ferns and allies found in Hawaiʻi. These DNA and corresponding herbarium specimens are publicly available upon request and have been used in a variety of research projects, including studies on the phylogenetics of native species (Howarth et al., 1997, 2003; Iwanycki Ahlstrand et al., 2019), leaf fungal diversity (Datlof et al., 2017), and diversification patterns of endemic species across the island archipelago (Hobbs and Baldwin, 2013; Yang et al., 2018).

Here, we set out to understand the effects of age and storage method (herbarium vs. DNA banked in a freezer) on DNA quality for paired samples in the HPDL and the Joseph F. Rock Herbarium at the University of Hawaiʻi (Honolulu, Hawaiʻi, USA). In particular, we focused on (1) sequencing taxa that are endemic to Hawaiʻi to generate new genomic resources from low‐coverage genome skimming and (2) comparing library quality and sequencing results from specimens that, when collected, were simultaneously accessioned in the HPDL and the herbarium. By using these paired herbarium tissue and frozen DNA specimens, we remove any genotype‐ or collection‐specific anomalies and can directly infer effects of storage method and age. We find that while both storage methods provide DNA of a quality suitable for high‐throughput sequencing, freezer‐stored DNA samples are less degraded over time.

## METHODS

### Hawaiian Plant DNA Library sampling

Hawaiian Plant DNA Library samples in this study were collected between 1994 and 2019. Upon collection, samples were accessioned in the Joseph F. Rock Herbarium (Appendix 1). Fresh tissue for DNA extraction was field collected into a plastic bag and remained at 4°C until it was extracted via a modified cetyltrimethylammonium bromide (CTAB) protocol and cesium banded (Morden et al., 1996; Randell and Morden, 1999). Purified DNA was stored at −20°C until this analysis. Samples in this analysis were chosen to represent native Hawaiian species, to span a breadth of duration in cold storage, and when possible, to be paired with herbarium sheet tissue based on the original plant specimen that was deposited in the HPDL freezer collection.

### DNA extraction and library construction

Small amounts of herbarium specimen tissue were sampled (Appendix S1; see Supporting Information with this article) as input for a modified CTAB DNA extraction protocol (Doyle, 1987). Briefly, this tissue was ground with mortar and pestle in liquid nitrogen and incubated at room temperature in an extraction buffer containing 0.35 M sorbitol and 10 mM β‐mercaptoethanol to remove phenolic compounds from the plant tissue. The supernatant was removed and replaced with 300 μL of extraction buffer and 400 μL of CTAB lysis buffer, and the samples were then incubated for 1 h at 65°C. After chloroform and isoamyl alcohol phase separation, samples were chilled at −20°C overnight. All DNA samples (herbarium and HPDL) were quantified via the Qubit dsDNA Broad Range Assay Kit (Invitrogen, Waltham, Massachusetts, USA). Before library construction, all samples (both herbarium and HPDL) were 3X bead‐cleaned using homemade magnetic beads prepared from Sera‐Mag SpeedBeads (Thermo Fisher Scientific, Waltham, Massachusetts, USA) by the Microbial Genomics and Analytical Laboratory Core at the University of Hawaiʻi at Mānoa. Libraries were quantified again after bead‐cleaning via the Qubit dsDNA Broad Range Assay Kit. Using the KAPA HyperPlus library kit (Roche, Indianapolis, Indiana, USA), samples were enzymatically fragmented for 4 min before end repair, A‐tailing, and ligation per the manufacturer's protocol. The adapters used in the ligation reaction are Y‐stub adapters purchased from BadDNA lab at the University of Georgia (Athens, Georgia, USA) (Glenn et al., 2019). PCR was performed for eight cycles using dual‐indexed barcoded primers from the BadDNA lab. Samples were 0.8X bead‐cleaned prior to PCR to remove adapter dimers, and the PCR products were 1.0X bead‐cleaned and eluted into 10 mM Tris HCl for storage. Completed libraries were initially assessed via the Qubit dsDNA High Sensitivity Assay Kit, and then via qPCR using the KAPA Library Quantitation kit. All libraries were diluted to approximately 3 nM and then pooled using equal volumes before being submitted to Novogene (Sacramento, California, USA) for sequencing on a NovaSeq 6000 (Illumina, San Diego, California, USA) with 150‐bp paired‐end reads.

### Sequence analysis

All sequence data were first checked for base quality and the presence of short fragments using FastQC (Andrews, 2010). Trimmomatic v0.39 was used to quality trim the ends of reads and remove the presence of Illumina sequence adapters (Bolger et al., 2014). Paired reads that were retained post‐trimming were used as input for chloroplast genome assembly via the assembler GetOrganelle (Jin et al., 2020). Assembly read depth and average insert size were obtained from GetOrganelle output files, and assembly length and GC content were determined using SeqKit (Shen et al., 2016). For final plastid assemblies that consisted of multiple scaffolds, a weighted average was used to characterize GC content. Chloroplast assemblies were annotated for protein‐coding genes and ribosomal RNAs using GeSeq (Tillich et al., 2017). Raw sequencing reads and assemblies have been deposited in GenBank (Appendix 2).

**Figure 1:**
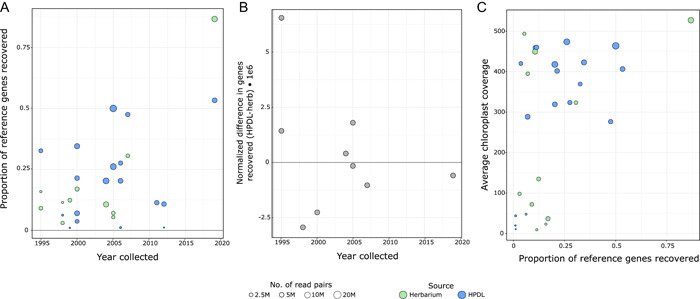
(A) Scatterplot showing the relationships between collection year and proportion of the 353 reference genes recovered per library, for samples derived from both the Hawaiian Plant DNA Library (HPDL) (blue) and herbarium tissue (green). Dot size indicates the overall sequencing effort for each library in total read pairs surviving Trimmomatic. (B) Scatterplot showing the difference in the number of the 353 reference genes recovered for paired libraries (i.e., samples collected at the same time and stored in both HPDL and herbarium conditions), normalized by total number of reads per library: (no. of reference genes HPDL/no. of reads) − (no. of reference genes herbarium/no. of reads). (C) Scatterplot showing the correlation between the proportion of reference genes recovered and total chloroplast assembly coverage per library, with sizes of dots scaled by the number of reads as in A.

In addition to analyzing the organellar sequences, we were interested in understanding the degree to which nuclear gene targets could be recovered. We used HybPiper (Johnson et al., 2016) to map and assemble reads corresponding to the Angiosperms353 single‐copy nuclear gene bait set (Johnson et al., 2019), or in the case of *Cibotium menziesii* Hook., from the GoFlag 451 bait set (Breinholt et al., 2021). Reference sequences of the 353 genes were downloaded from the Kew Tree of Life Explorer (https://treeoflife.kew.org/); for each genus we sequenced, we attempted to download references from a congeneric species when available, but resorted to closely related genera (e.g., within the family) otherwise (Appendix 1). For *Cibotium*, assembled sequences of the GoFlag 451 loci from the closest relative in the Cyatheales (*Thyrsopteris elegans* Kunze) were used as a reference; due to low read mapping rates, no nuclear genes were recovered for *Cibotium* and it was not further analyzed in this context. HybPiper was run with default settings but with coverage for SPAdes lowered to 4 (‐‐cov_cutoff). The total number of assembled contigs across sample type, age of specimen, and relative to sequencing output was evaluated across libraries (Figure 1). Analysis of the effects of storage method, collection year, and number of sequencing reads on the number of reference nuclear genes recovered was performed using ANOVA in R version 4.2.1 (R Core Team, 2019). Additionally, we sought to determine whether the genome sizes of these species may have affected the proportion of single‐copy nuclear genes recovered. To address this, we obtained genome size estimates from the Kew Plant C‐value Database (https://cvalues.science.kew.org/). Depending on the data available, we either obtained the average genome size of the relevant genus, or the relevant family if no plants had been sampled within the genus (Appendix S2). Herbarium and HPDL samples were analyzed separately to determine whether the proportion of single‐copy nuclear genes recovered was correlated to the average genome size of the most relevant clade using a Pearson correlation coefficient in R version 4.2.1 (R Core Team, 2019).

Herbarium specimens, much like ancient DNA, are subject to DNA degradation most commonly via DNA deamination (Staats et al., 2011; Weiß et al., 2016). To determine the extent to which storage in herbaria affects the DNA within plant cells, we compared herbarium libraries and their freezer‐stored DNA counterparts via read mapping to the chloroplast genome assemblies. For each species, reads were mapped from both herbarium and HPDL samples to the more complete HPDL‐assembled chloroplast using Bowtie2 (Langmead and Salzberg, 2012). Mapped reads were sorted using SAMtools (Li and Durbin, 2009), and polymorphisms were called and filtered using BCFtools (Danecek et al., 2021). Positions with quality <20 were removed. Heterozygous C‐to‐T substitutions were inferred to be areas of putative deamination, as it is unlikely for all homologous nucleotides to undergo the deamination process. The number of C‐to‐T heterozygous sites was determined for both the herbarium tissues and freezer‐stored DNA samples and was used to calculate a proportion of putative deaminated sites relative to the number of all heterozygous loci. The proportion of putative deamination polymorphisms was compared between paired (herbarium/HPDL) libraries via a χ^2^ test with one degree of freedom in instances where both libraries had at least 10 polymorphisms with which to establish a proportion (only possible in *Acacia koa* A. Gray and *Carex wahuensis* C. A. Mey.). The number and proportion of putative deaminated sites were correlated to sampling year in the herbarium tissues and HPDL libraries separately in R version 4.2.1 (R Core Team, 2019).

## RESULTS

### Sample and library quality

Many herbarium tissue extractions had higher initial DNA concentrations than their cold storage DNA bank counterparts. However, as we normalized input DNA amounts into library construction, the resulting DNA concentrations of the sequencing libraries were equivalent (Appendix S1).

### Chloroplast genome assembly

Herbarium tissue library samples had significantly smaller insert sizes of mapped chloroplast reads compared to their freezer‐stored DNA paired samples, taking into account covariates of read numbers and year (*F*_2,24_ = 112.864, *P* < 0.001). There was also a significant interaction effect between library size and sampling year (*F*_1,24_ = 7.392, *P* < 0.05). Similarly, herbarium tissue samples also had higher amounts of adapter sequences in the reads (*F*_2,24_ = 43.229, *P* < 0.001), with sampling year a significant covariate in the model (*F*_1,24_ = 4.776, *P* < 0.05). The ability to assemble a complete circular chloroplast genome was affected by herbarium tissue storage in a genus‐specific manner. For example, in *Acacia koa* the herbarium tissue library was assembled into 26 scaffolds compared to the frozen DNA stored library that assembled into a single linear fragment. Furthermore, the assembly length of the herbarium tissue sample for *A. koa* was more than 54,000 bp shorter and had reduced mapping coverage (Table 1). Similarly, the *Deschampsia nubigena* Hillebr. herbarium tissue sample produced an assembly of 11 scaffolds compared to the frozen DNA stored library that assembled into a circular molecule (Table 1). Interestingly, despite the disparity in *D. nubigena* assemblies, the lengths were similar, with the herbarium tissue sample actually being longer despite having reduced mapping coverage. In contrast to *Acacia* and *Deschampsia*, many other genera exhibited remarkable consistency between herbarium tissue and frozen DNA stored samples in terms of assembly completeness and length. For example, comparisons between storage types yielded identically sized circular genomes in *Erythrina sandwicensis* O. Deg., *Santalum ellipticum* Gaudich. (from 1995), and *Sesbania tomentosa* Hook. & Arn., and nearly identical lengths in *Carex wahuensis*, *Clermontia fauriei* H. Lév., *Clermontia kakeana* Meyen, and *S. ellipticum* (from 2007) (Table 1).

**Table 1:** Source information for DNA samples, chloroplast assembly, and sequencing library quality metrics. Full accession information is available in Appendix 1.

Species	Type	Year	Chloroplast assembly^a^	Length (bp)	Coverage^b^	Insert size^c^ (bp)	% pairs surviving^d^	% adapter^e^	Prop. nuclear^f^
*Acacia koa*	HAW	1998	26	121,248	9.30	190.35	34.17	32.21	0.11
*A. koa*	HPDL	1998	1^g^	175,435	47.70	327.77	90.10	5.26	0.06
*Carex wahuensis*	HAW	2019	17	125,940	527.30	317.64	91.41	5.43	0.87
*C. wahuensis*	HPDL	2019	17	126,612	406.60	364.19	79.58	3.44	0.53
*C. wahuensis* subsp. *wahuensis*	HPDL	2006	16	128,174	323.70	314.21	87.83	6.25	0.28
*Cibotium menziesii*	HPDL	1994	2	156,865	34.60	337.34	71.59	4.01	n.a.^h^
*Clermontia arborescens*	HPDL	2011	1	164,866	460.10	308.00	89.77	5.11	0.11
*Clermontia fauriei*	HAW	2005	Circular	165,082	395.00	210.88	58.19	29.86	0.07
*C. fauriei*	HPDL	2005	Circular	165,214	464.00	354.72	90.64	6.42	0.5
*Clermontia kakeana*	HAW	2005	Circular	164,645	493.70	229.64	62.67	24.72	0.05
*C. kakeana*	HPDL	2005	Circular	164,717	473.80	330.95	91.88	5.28	0.26
*C. kakeana*	HPDL	2012	Circular	163,907	459.00	330.84	90.77	5.28	0.11
*Cyanea calycina*	HPDL	2000	Circular	165,198	420.20	304.25	90.90	5.91	0.04
*Cyanea spathulata*	HAW	1995	15	139,680	23.20	199.96	36.49	47.63	0.16
*Deschampsia nubigena*	HAW	2000	11	154,034	36.70	210.72	70.57	19.96	0.17
*D. nubigena*	HPDL	2000	Circular	152,939	288.70	340.71	93.71	3.71	0.07
*Erythrina sandwicensis*	HAW	1998	1	152,145	98.10	216.13	64.77	25.73	0.03
*E. sandwicensis*	HAW	2004	Circular	152,399	449.60	251.54	76.83	16.56	0.11
*E. sandwicensis*	HPDL	2004	Circular	152,399	417.90	334.34	90.37	5.44	0.23
*Peperomia cookiana*	HPDL	2000	1	151,147	401.80	322.92	87.37	6.83	0.21
*P. cookiana*	HPDL	2012	22	124,590	10.80	323.53	30.58	4.78	0.01
*Peperomia latifolia*	HPDL	2000	Circular	151,016	423.00	320.97	89.64	7.08	0.35
*Pritchardia hillebrandii*	HPDL	2006	Circular	157,175	319.30	335.61	90.72	5.80	0.20
*Santalum ellipticum*	HAW	1995	Circular	144,330	72.00	251.71	59.20	29.87	0.09
*S. ellipticum*	HPDL	1995	Circular	144,330	369.60	339.99	90.79	5.86	0.33
*S. ellipticum*	HAW	2007	Circular	144,540	323.50	214.72	70.94	20.44	0.31
*S. ellipticum*	HPDL	2007	Circular	144,526	276.60	330.50	84.10	5.24	0.48
*Sesbania tomentosa*	HAW	1999	Circular	157,141	134.70	201.73	67.51	23.05	0.12
*S. tomentosa*	HPDL	1999	Circular	157,141	19.80	337.55	77.48	4.58	0.01
*Sida fallax*	HPDL	2006	Circular	160,058	43.80	332.54	92.25	4.03	0.01

*Note*: HPDL = sample from the Hawaiian Plant DNA Library; HAW = sample from the Joseph F. Rock Herbarium, University of Hawaiʻi (Honolulu, Hawaiʻi, USA).

^a^ For assembly, “circular” is reported if a complete molecule was assembled; otherwise, the number of scaffolds is reported.

^b^ Coverage of the chloroplast assembly.

^c^ Insert size refers to GetOrganelle output, which infers average insert size based on mapped paired reads.

^d^ Read pairs surviving Trimmomatic.

^e^ The percent of reads removed by FastQC due to adapter readthrough.

^f^ The proportion of the 353 reference genes recovered; reference used per species is available in Appendix 1.

^g^ A single scaffold was at times recovered, but was not circular.

^h^ *Cibotium* was excluded from nuclear analysis because the study focused on Angiosperms353 gene sets.

### Nuclear recovery and DNA deamination

The largest predictors of proportion of the 353 reference genes recovered in our sequencing libraries were the sheer number of paired reads that went into the HybPiper analysis (*F*_1,24_ = 23.451, *P* < 0.001), the collection year of the starting material (*F*_1,24_ = 8.494, *P* < 0.01), and the interaction of both year and read number (*F*_1,24_ = 10.563, *P* < 0.01). Whether a sample was from herbarium tissue or HPDL was not significant in predicting nuclear gene recovery (Figure 1A). Furthermore, there was no correlation between the difference in number of genes recovered between paired HPDL and herbarium tissue samples across time (Figure 1B). Finally, chloroplast coverage and the proportion of nuclear genes recovered were correlated (*P* = 0.008), but both are clearly influenced by the total number of reads (Figure 1C). The proportion of single‐copy nuclear genes exhibited a negative, but non‐significant, correlation with genome size for both the herbarium (*r* = −0.26, *P* = 0.58) and HPDL samples (*r* = −0.37, *P* = 0.29).

Chloroplast polymorphisms were variable between herbarium tissue and frozen DNA stored samples. The *Acacia koa* herbarium tissue library did not have a significantly higher proportion of putative deaminating polymorphisms than the frozen DNA stored library (χ^2^ = 0.322, *P* > 0.05, *df* = 1). *Carex wahuensis* also showed no significant difference in deamination between storage methods (χ^2^ = 1.941, *P* > 0.05, *df* = 1). For almost all other paired comparisons, the frozen DNA stored library had too few heterozygous locations to establish baseline proportions for a χ^2^ analysis. For example, in *Deschampsia nubigena* the herbarium tissue sample had 36 putative deamination sites out of 139 heterozygous sites total, in contrast to just one out of four sites in the frozen DNA stored library (Appendix S3). In two separate herbarium tissue/frozen DNA paired comparisons within the same species (*Santalum ellipticum*), both frozen DNA samples had zero heterozygous sites while the paired herbarium tissue samples had three and six putative deamination sites, respectively. In only one comparison (*Sesbania tomentosa*) did the frozen DNA stored sample have more heterozygous sites than the herbarium tissue sample (26 vs. 1), although none of the 26 represented putative deamination C‐to‐T substitutions (Appendix S3). Although sample sizes were low, no relationship was found for herbarium tissue samples between the number or proportion of putative deaminated sites with age (all tests *P* > 0.05), a pattern that was also found in the frozen DNA stored libraries (all tests *P* > 0.05).

## DISCUSSION

### New genetic resources in Hawaiian taxa

The Hawaiian flora is highly endemic and threatened; of the estimated 1400 native plant species, 90% are endemic and nearly a quarter are listed as threatened or endangered (Department of Land and Natural Resources, 2023; Pacific Islands Fish and Wildlife Office, 2023). The chloroplast genomes assembled here represent the first significant genomic resource for many of these taxa. For a few taxa, an appreciable number of nuclear genes were also recovered (Table 1). We hope these new data can be the starting point for future projects in island biogeography, evolution, and rare plant conservation.

### Effect of herbarium storage on plastid and nuclear genome recovery

Sequencing libraries generated from herbarium tissue had high proportions of adapter readthrough during Illumina sequencing, likely as a result of more highly degraded DNA. Despite this noticeable difference, many chloroplast genomes generated from herbarium tissue were recovered in full circular molecules, with a complete circular plastid genome being assembled from a herbarium tissue sample that was 27 years old. However, in some paired comparisons, the herbarium tissue sample assembly was incomplete relative to the assembly from the frozen DNA stored sample. In *Acacia koa* and *Deschampsia nubigena*, the sequencing libraries generated from herbarium tissue samples had chloroplast assemblies made up of 26 and 11 scaffolds, respectively, compared to the frozen DNA stored samples, for which chloroplast assemblies were in a single molecule (linear in the case of *A. koa*, circular in the case of *D. nubigena*). It is likely that there is a species‐specific effect on DNA fragmentation, in addition to the effects of storage method and age. It is already established that inhibitory secondary compounds are affecting herbarium‐based DNA extractions in particular species (Marinček et al., 2022). Furthermore, there could be an environmental factor, in that the original collections were made under varying conditions and may have been processed in slightly different ways for herbarium accessioning. Both the herbarium tissue samples and the HPDL samples displayed a non‐significant trend in which species with smaller genomes had a higher proportion of nuclear single‐copy genes recovered. Unfortunately, the relatively small size of our data set, paired with limited sampling in public databases, precluded us from exploring this relationship further in this work. The effects of genome size and/or ploidy on herbarium genomic studies should be an important consideration for future studies, as it may partially contribute to why certain taxa routinely yield less DNA after being stored on a herbarium sheet.

### Effect of herbarium storage on deamination

DNA from herbarium tissue specimens has been noted to degrade, either due to the preservation process or slowly over time (Staats et al., 2011). Much of this degradation appears to happen in plastid genomes and results in the conversion of C bases to T bases as a result of cytosine deamination. There was no clear pattern of increasing deamination in herbarium tissue specimens compared to their frozen DNA counterparts, nor was there a clear pattern across time. In the two species that we could statistically assess (*Acacia koa* from 1998 and *Carex wahuensis* from 2019), neither had a significantly higher number of putative deamination sites in the herbarium tissue sample compared to the frozen DNA sample. This could be a function of the short amount of time since the collection of these specimens, resulting in minimal accumulation of spontaneous mutations in the herbarium tissue samples. DNA extracted from older herbarium tissues (1995) of *Santalum ellipticum* had a larger number (four vs. two) of putative deamination sites compared to a younger specimen (2007); however, the proportions of putative deamination sites were similar between these samples. Notably, no evidence of deamination was seen in either of the HPDL samples of these *S. ellipticum* specimens (1995 and 2007). Additionally, we can deduce that either lineage‐specific or preparation‐specific effects are likely at play as no evidence of deamination was seen in two herbarium tissue specimens of *Clermontia* from 2005. Previous work found that more than 20% of mutated sites were putatively due to deamination, representing a sizable fraction of polymorphism arising from stochastic rather than evolutionary processes (Staats et al., 2011). Here, the levels of polymorphism introduced by deamination were much lower, but also non‐zero, and could feasibly affect phylogenetic or population genetic inference of slowly evolving plastid genomes.

### Conclusions

Chloroplast assemblies and, to a lesser extent, nuclear genes, can be recovered from both herbarium‐stored tissues and frozen DNA samples. In general, the frozen DNA stored libraries were of higher quality (e.g., less fragmented, higher coverage), but age and sequencing effort had significant effects on the resulting chloroplast assembly. Both herbaria‐stored tissues and frozen DNA samples face potential pitfalls in terms of storage; the former is susceptible to pests and environmental conditions, while the latter can undergo catastrophic equipment failures. Using both storage methods—particularly for rare and threatened floras—can represent an insurance policy for future studies. Moreover, while herbarium tissue–derived DNA has proven time and again to be useful in exploring a number of research questions, for studies requiring larger fragments from more intact DNA, freezer‐stored DNA libraries will be invaluable.

## AUTHOR CONTRIBUTIONS

E.V.M. conceived of the study, analyzed the data, and wrote the manuscript; C.D. assisted with laboratory work including DNA extractions; M.Y. and C.M. provided HPDL samples and feedback on the manuscript; K.H. assisted with laboratory work and data analysis and helped to write the final manuscript. All authors approved the final version of the manuscript.

## Supporting information


**Appendix S1**. Information on the weight of tissue used for DNA and all pre‐ and post‐library quantification.

**Appendix S2**. Genome size estimates for the taxa studied.

**Appendix S3**. DNA deamination results per library.

## Data Availability

Raw sequencing reads are available via the National Center for Biotechnology Information (BioProject PRJNA911344); complete chloroplast assemblies are available from GenBank (Appendix 2).

## APPENDIX 1 Herbarium voucher numbers and Hawaiian Plant DNA Library (HPDL) accession numbers.^a^

**Table:**

Species name ‐ Latin	Species name ‐ ʻōlelo	Year	Accession no.
*Acacia koa*	koa	1998	HAW44000
*Acacia koa*	koa	1998	HPDL1738
*Carex wahuensis*		2019	HAW47147
*Carex wahuensis*		2019	HPDL10794
*Carex wahuensis* subsp. *wahuensis*		2006	HPDL5367
*Cibotium menziesii*	hāpu‘u	1994	HPDL614
*Clermontia arborescens*	hāhā, ‘ōhā wai	2011	HPDL6871
*Clermontia fauriei*	hāhā, ‘ōhā wai	2005	HAW43875
*Clermontia fauriei*	hāhā, ‘ōhā wai	2005	HPDL5095
*Clermontia kakeana*	hāhā, ‘ōhā wai	2005	HAW43369
*Clermontia kakeana*	hāhā, ‘ōhā wai	2005	HPDL4919
*Clermontia kakeana*	hāhā, ‘ōhā wai	2012	HPDL7023
*Cyanea calycina*	hāhā	2000	HPDL3068
*Cyanea spathulata*	hāhā	1995	HAW43878
*Deschampsia nubigena*		2000	HAW44234
*Deschampsia nubigena*		2000	HPDL2834
*Erythrina sandwicensis*	wiliwili	1998	HAW44007
*Erythrina sandwicensis*	wiliwili	2004	HAW44008
*Erythrina sandwicensis*	wiliwili	2004	HPDL4393
*Peperomia cookiana*	‘ala‘ala wai nui	2000	HPDL2811
*Peperomia cookiana*	‘ala‘ala wai nui	2012	HPDL7125
*Peperomia latifolia*	‘ala‘ala wai nui	2000	HPDL2812
*Pritchardia hillebrandii*	loulu	2006	HPDL5488
*Santalum ellipticum*	‘iliahi	1995	HAW44434
*Santalum ellipticum*	‘iliahi	1995	HPDL340
*Santalum ellipticum*	‘iliahi	2007	HAW44435
*Santalum ellipticum*	‘iliahi	2007	HPDL5587
*Santalum tomentosa*	‘iliahi	1999	HAW44013
*Sesbania tomentosa*	‘ohai	1999	HPDL2110
*Sida fallax*	‘ilima	2006	HPDL5524

^a^ Reference species for the sample species are: *Acacia koa* = *Acacia pycnantha*; *Carex wahuensis* = *Carex duriuscula*; *Clermontia arborescens* = *Adenophora polyantha*; *Clermontia fauriei* = *Adenophora polyantha*; *Clermontia kakeana* = *Adenophora polyantha*; *Cyanea calycina* = *Adenophora polyantha*; *Cyanea spathulata* = *Adenophora polyantha*; *Deschampsia nubigena* = *Deschampsia cespitosa*; *Erythrina sandwicensis* = *Erythrina lysistemon*; *Peperomia cookiana* = *Peperomia fraseri*; *Peperomia latifolia* = *Peperomia fraseri*; *Pritchardia hillebrandii* = *Pritchardia arecina*; *Santalum ellipticum* = *Santalum album*; *Sesbania tomentosa* = *Sesbania cannabina*; *Sida fallax* = *Sida acuta*. No reference species was used for *Cibotium menziesii*.

## APPENDIX 2 GenBank and Sequence Read Archive (SRA) IDs.

**Table:**

SequenceID	Species	Accession no.	GenBank	SRA BioSample	BioProject
hpdl1	*Acacia koa*	HAW44000		SAMN32180107	PRJNA911344
hpdl10	*Clermontia kakeana*	HAW43369		SAMN32180108	PRJNA911344
hpdl11	*Clermontia kakeana*	HPDL4919	OQ870892	SAMN32180109	PRJNA911344
hpdl12	*Clermontia kakeana*	HPDL7023		SAMN32180110	PRJNA911344
hpdl13	*Cyanea calycina*	HPDL3068	OQ870893	SAMN32180111	PRJNA911344
hpdl14	*Cyanea spathulata*	HAW43878		SAMN32180112	PRJNA911344
hpdl15	*Deschampsia nubigena*	HAW44234		SAMN32180113	PRJNA911344
hpdl16	*Deschampsia nubigena*	HPDL2834	OQ870894	SAMN32180114	PRJNA911344
hpdl17	*Erythrina sandwicensis*	HAW44007		SAMN32180115	PRJNA911344
hpdl18	*Erythrina sandwicensis*	HAW44008		SAMN32180116	PRJNA911344
hpdl19	*Erythrina sandwicensis*	HPDL4393	OQ870895	SAMN32180117	PRJNA911344
hpdl2	*Acacia koa*	HPDL1738	OQ870901	SAMN32180118	PRJNA911344
hpdl20	*Peperomia cookiana*	HPDL2811	OQ870896	SAMN32180119	PRJNA911344
hpdl21	*Peperomia cookiana*	HPDL7125		SAMN32180120	PRJNA911344
hpdl22	*Peperomia latifolia*	HPDL2812	OQ870897	SAMN32180121	PRJNA911344
hpdl23	*Prtichardia hillebrandii*	HPDL5488	OQ870898	SAMN32180122	PRJNA911344
hpdl24	*Santalum ellipticum*	HAW44435		SAMN32180123	PRJNA911344
hpdl25	*Santalum ellipticum*	HAW44434		SAMN32180124	PRJNA911344
hpdl26	*Santalum ellipticum*	HPDL340		SAMN32180125	PRJNA911344
hpdl27	*Santalum ellipticum*	HPDL5587	OQ870899	SAMN32180126	PRJNA911344
hpdl28	*Sesbania tomentosa*	HAW44013		SAMN32180127	PRJNA911344
hpdl29	*Sesbania tomentosa*	HPDL2110	OQ870900	SAMN32180128	PRJNA911344
hpdl3	*Carex wahuensis*	HAW47147		SAMN32180129	PRJNA911344
hpdl30	*Sida fallax*	HPDL5524	OQ870902	SAMN32180130	PRJNA911344
hpdl4	*Carex wahuensis*	HPDL10794		SAMN32180131	PRJNA911344
hpdl5	*Carex wahuensis* subsp. *wahuensis*	HPDL5367		SAMN32180132	PRJNA911344
hpdl6	*Cibotium menziesii*	HPDL614		SAMN32180133	PRJNA911344
hpdl7	*Clermontia arborescens*	HPDL6871	OQ870903	SAMN32180134	PRJNA911344
hpdl8	*Clermontia fauriei*	HAW43875		SAMN32180135	PRJNA911344
hpdl9	*Clermontia fauriei*	HPDL5095	OQ870904	SAMN32180136	PRJNA911344
